# β-Lactamase Tools for Establishing Cell Internalization and Cytosolic Delivery of Cell Penetrating Peptides

**DOI:** 10.3390/biom8030051

**Published:** 2018-07-11

**Authors:** Shane R. Stone, Tatjana Heinrich, Suzy M. Juraja, Jiulia N. Satiaputra, Clinton M. Hall, Mark Anastasas, Anna D. Mills, Christopher A. Chamberlain, Scott Winslow, Kristin Priebatsch, Paula T. Cunningham, Katrin Hoffmann, Nadia Milech

**Affiliations:** 1 Telethon Kids Institute, University of Western Australia, Subiaco, WA 6008, Australia; shane.stone@telethonkids.org.au (S.R.S.); tatjana.heinrich@telethonkids.org.au (T.H.); suzy.juraja@telethonkids.org.au (S.M.J.); jiulia.satiaputra@telethonkids.org.au (J.N.S.); clinton.hall@telethonkids.org.au (C.M.H.); mark.anastasas@telethonkids.org.au (M.A.); anna.mills@telethonkids.org.au (A.D.M.); chrisa.chamberlain@outlook.com (C.A.C.); scott.winslow@telethonkids.org.au (S.W.); kristin.priebatsch@telethonkids.org.au (K.P.); paula.cunningham@telethonkids.org.au (P.T.C.); katrin.hoffmann@telethonkids.org.au (K.H.); 2 Phylogica Pty Ltd., Subiaco, WA 6008, Australia

**Keywords:** cell penetrating peptide, high-throughput screening, functional validation, cell internalization, cytosolic delivery

## Abstract

The ability of cell penetrating peptides (CPPs) to deliver biologically relevant cargos into cells is becoming more important as targets in the intracellular space continue to be explored. We have developed two assays based on CPP-dependent, intracellular delivery of TEM-1 β-lactamase enzyme, a functional biological molecule comparable in size to many protein therapeutics. The first assay focuses on the delivery of full-length β-lactamase to evaluate the internalization potential of a CPP sequence. The second assay uses a split-protein system where one component of β-lactamase is constitutively expressed in the cytoplasm of a stable cell line and the other component is delivered by a CPP. The delivery of a split β-lactamase component evaluates the cytosolic delivery capacity of a CPP. We demonstrate that these assays are rapid, flexible and have potential for use with any cell type and CPP sequence. Both assays are validated using canonical and novel CPPs, with limits of detection from <500 nM to 1 µM. Together, the β-lactamase assays provide compatible tools for functional characterization of CPP activity and the delivery of biological cargos into cells.

## 1. Introduction

β-Lactamase is used extensively as an independent intracellular reporter in mammalian cell assays; for example, in tracking signaling pathways [1,2,3] or viral fusions [4]. These assays use a fluorescence resonance energy transfer (FRET)-based substrate, CCF2-AM, which is specifically developed for *Escherichia coli* TEM-1 β-lactamase [5,6,7]. CCF2-AM is rapidly taken up by cells, where it is de-esterified by endogenous cytoplasmic esterases. This converts CCF2-AM into negatively charged CCF2, which is trapped in the cytoplasm. Any β-lactamase in the cytosol can then rapidly cleave CCF2, disrupt the FRET, and produce a signal shift from green to blue fluorescence. The cell-type independence and utility in the cytosolic environment make the combination of CCF2-AM and β-lactamase an ideal reporter tool for assessing the functional activity of cell penetrating peptides (CPPs).

Cell penetrating peptides are peptides that can cross cellular membranes, facilitating the delivery of a variety of molecular cargos to the inside of cells both in vitro and in vivo. Such cargos include nanoparticles, small molecules, and biologics; for example, large proteins, small peptides and oligonucleotides [8]. Cell penetrating peptides also embody many ideal features that characterize a desirable intracellular delivery vehicle, including minimal cell perturbation, scalability and dosage control [9]. The more efficiently the delivery vehicle can permeate the cell wall and internalize into cells, the more effective the intracellular delivery of active cargos to the cytosol and nucleus will be. This makes CPPs of particular interest as modern drug discovery programs increase their focus on the intracellular target space. Identifying CPPs with potent intracellular internalization activity at therapeutically relevant concentrations is a key goal of CPP discovery research and therapeutic programs.

A variety of assays have been developed to detect CPP uptake. Those with fluorescence-based readouts are particularly amenable to rapid screening in high-throughput format. However, conjugating CPPs with small molecule fluorophore cargos may not permit discrimination between internalized and endosomally-trapped CPPs without the inclusion of additional experimental variables such as endocytic inhibitors or markers; moreover, such labeling can also result in fluorophore-dependent changes to CPP function [10,11,12,13]. Split-protein fluorophore assays definitively discriminate between cytoplasmic delivery and endosomal entrapment, but also require longer incubation for the folding of the fluorophore [14,15]. Hence, there remains a need for a simple, rapid, sensitive, easily-visualized assay for CPP internalization that has a minimal background, is broadly applicable to any cell type, and is compatible with high-throughput fluorescence techniques.

Here, we present two new tools using β-lactamase as a reporter for internalization and cytosolic delivery. First, we have engineered a β-lactamase uptake assay which measures CPP-dependent enzyme internalization into mammalian cells. This assay is compatible with both synthetic CPPs conjugated to β-lactamase, or recombinant CPP-β-lactamase fusion proteins. Second, we have developed a split β-lactamase assay, where the complementation and formation of the functional β-lactamase molecule can only occur after cytosolic delivery of a CPP-cargo fusion protein. Together, these assays provide versatile tools for identifying and understanding the properties of potent CPPs.

## 2. Results

### 2.1. β-Lactamase Internalisation Assay

**Figure 1:**
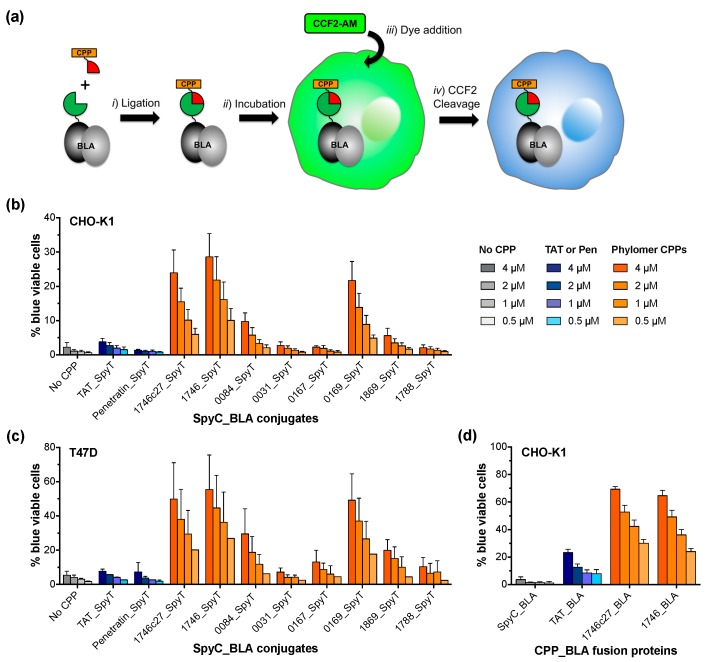
A cell penetrating peptide (CPP)- mediated β-lactamase internalization assay. (**a**) Recombinant β-lactamase can be delivered into cells when conjugated to CPPs, for example through SpyTag/SpyCatcher conjugation chemistry (step *i*). The internalization of β-lactamase (step *ii*) is detected after the addition of dye substrate (step *iii*) through cleavage of cytosol-retained CCF2 substrate (step *iv*), which is measured via a change in fluorescence signal from green (520 nm) to blue (450 nm); (**b**) The internalization of CPP-conjugated SpyC_β-lactamase (SpyC_BLA) in CHO-K1 cells is dose-dependent, resulting in an increase in the percentage of blue viable cells as estimated by flow cytometry. Increased fluorescence is indicative of greater cytoplasmic delivery capacity of the CPP. β-Lactamase is conjugated to conventional CPPs TAT_SpyT (*n* = 4) and Penetratin_SpyT (*n* = 2), as well as phylomer CPPs 1746c27_SpyT (*n* = 7), 1746_SpyT (*n* = 5), 0084_SpyT (*n* = 6), and 0031_SpyT, 0167_SpyT, 0169_SpyT, 1869_SpyT, and 1788_SpyT (*n* = 3). Unconjugated SpyC_BLA does not internalize (‘No CPP’ negative control; *n* = 7). Error bars represent standard error of the mean of *n* independent experiments (duplicate samples within each experiment); (**c**) The internalization of CPP-conjugated SpyC_BLA in T47D cells is also dose-dependent, resulting in an increase in the percentage of blue viable cells as estimated by flow cytometry. β-Lactamase is conjugated to conventional CPPs TAT_SpyT and Penetratin_SpyT (*n* = 1), as well as Phylomer CPPs 1746c27_SpyT, 1746_SpyT, 0084_SpyT, 0031_SpyT, 0167_SpyT, 0169_SpyT, 1869_SpyT, and 1788_SpyT (*n* = 3). Unconjugated SpyC_BLA does not internalize (No CPP negative control; *n* = 3). Error bars represent standard error of the mean of *n* independent experiments (duplicate samples); (**d**) The internalization of recombinantly expressed CPP_β-lactamase fusion protein (CPP_BLA) is dose-dependent, resulting in an increase in the percentage of blue viable cells as estimated by flow cytometry. β-Lactamase is expressed fused to conventional CPP TAT (*n* = 3), as well as Phylomer CPPs 1746c27 and 1746 (*n* = 2). SpyC_BLA (negative control) does not internalize. Error bars represent standard error of the mean of *n* independent experiments (CPP samples in triplicate; SpyC_BLA samples in triplicate (*n* = 1) and duplicate (*n* = 2)).

The β-lactamase internalization assay is based on the simple principle that CPP-dependent intracellular delivery of recombinant β-lactamase protein can be detected by cleavage of the CCF2 substrate in the cytosol (Figure 1a). In brief, CPP sequences and the β-lactamase cargo protein can be brought together by conjugation or can be recombinantly expressed as a CPP_β-lactamase (CPP_BLA) fusion (Figure 1a, step *i*). The CPP_BLA protein fusions or conjugates are incubated on cells, which are then washed to remove excess protein that has not internalized (Figure 1a, step *ii*). The cells are incubated further (i.e., loaded) with the CCF2-AM cell-permeable dye for 1 h (Figure 1a, step *iii*). Once inside the cell, CCF2-AM is converted by endogenous cytoplasmic esterases to CCF2, which is retained in the cytosol. In cells where β-lactamase has internalized through CPP activity, CCF2 is cleaved by β-lactamase (Figure 1a, step *iv*). This reaction is measured as a shift in fluorescent signal from green to blue. Signal conversion continues as long as there is CCF2 substrate remaining inside the cell.

To validate the assay, we first used a conjugation approach based on the SpyTag (SpyT) peptide/SpyCatcher (SpyC) protein ligation technology [16]. Thus, CPP sequences synthesized as SpyTag fusions (CPP_SpyT; sequences in Table A1), can be conjugated to recombinantly expressed SpyCatcher_β-lactamase protein (SpyC_BLA; sequence in Table A2) through formation of the SpyT/SpyC isopeptide bond. We conjugated SpyC_BLA to canonical CPPs TAT and Penetratin, as well as novel CPP sequences discovered from phylomer peptide libraries in our laboratory (manuscript submitted). Protein conjugates were incubated on CHO-K1 or T47D cells, measuring the fluorescence shift as percentage of blue fluorescent CHO-K1 or T47D cells after 1 h (Figure 1b,c, respectively). Successful internalization of β-lactamase was dose- and CPP-dependent, with a limit of detection of 500 nM for conjugated protein. The potency of TAT and Penetratin CPPs was comparable to those measured by independent split-GFP complementation assays [14].

**Figure 2:**
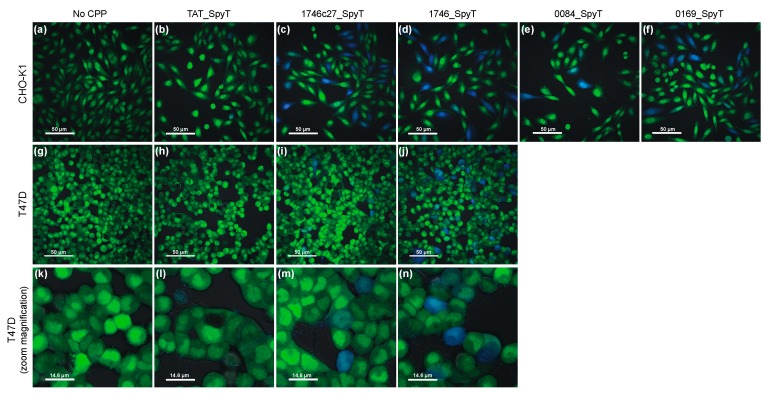
Live-cell confocal microscopy visualizes CPP-mediated β-lactamase internalization. The CPP-mediated uptake of CPP_SpyT/SpyC_BLA conjugates (4 µM) was visualized in CHO-K1 cells (**a**–**f**) and T47D cells (**g**–**n**). SpyC_BLA was conjugated to CPP_SpyT peptides. Unconjugated SpyC_BLA (No CPP negative controls) shows minimal evidence of internalization in both CHO-K1 and T47D cells (**a**,**g**,**k**). Conjugation with TAT_SpyT (**b**,**h**,**l**) or phylomer CPPs 1746c27_SpyT (**c**,**i**,**m**), 1746_SpyT (**d**,**j**,**n**), 0084_SpyT (**e**), and 0169_SpyT (**f**) enabled CPP-dependent uptake of SpyC_BLA. The degree of uptake is CPP-dependent and visualized by blue fluorescing cells. Bar scale is 50 µm (**a**–**j**) or 14.6 µm (**k**–**n**).

The two phylomer CPPs with the strongest β-lactamase internalization signal and the canonical CPP TAT (positive control) were expressed as direct fusions with β-lactamase (CPP_BLA proteins; sequences in Table A2). Internalization of these recombinant proteins was assessed in CHO-K1 cells using the β-lactamase assay. Uptake of CPP_BLA proteins recapitulated the dose- and CPP-dependent internalization trends seen with the CPP_SpyT/SpyC_BLA conjugates (Figure 1d). This variation of our internalization assay showed an increase in sensitivity, with a limit of detection of <500 nM for recombinant CPP_BLA proteins. The increased delivery of recombinant 1746_BLA was also verified in immunoblot experiments detecting cytoplasmic delivery of β-lactamase by Phylomer CPPs (Figure S1). For TAT-conjugated β-lactamase, a band corresponding to the size of β-lactamase alone was detected in membrane fractions. This is consistent with reports that up to 90% of TAT-fused cargos may remain trapped within endosomes and are not released to the cytoplasm [17,18,19], and suggests there is some degradation in the endosome. Lastly, β-lactamase internalization was visually confirmed by confocal live cell fluorescence microscopy in both CHO-K1 and T47D cells treated with CPP_SpyT/SpyC_BLA conjugates (Figure 2a–j).

### 2.2. Split β-Lactamase Cytosolic Delivery Assay

β-Lactamase has previously been explored as a split enzyme for probing intracellular protein–protein interactions [20], DNA methylation [21] and protein aggregation [22]. The premise of these methods is the splitting of β-lactamase into two inactive components, the N-terminal sequence (N-BLA) and the C-terminal sequence (C-BLA). Only upon being tethered closely together by an intermolecular interaction or protein–protein ligation can the enzyme re-complement and β-lactamase activity be reinstated.

To develop a β-lactamase system for the definitive validation of cytosolic delivery, we engineered N-BLA and C-BLA fragments as fusions with SpyT and SpyC, respectively (N-BLA_SpyT and SpyC_C-BLA). SpyT/SpyC ligation enables specific covalent linkage of the split components when mixed together, thus reactivating β-lactamase activity. SpyC_C-BLA was selected for mammalian expression in stable cell lines, and N-BLA_SpyT was selected for CPP incorporation onto its N-terminus (CPP_N-BLA_SpyT). Constitutively expressed SpyC_C-BLA in the cytoplasm of stable cell lines can be complemented by CPP-delivered N-BLA_SpyT, thus forming functional β-lactamase.

**Figure 3:**
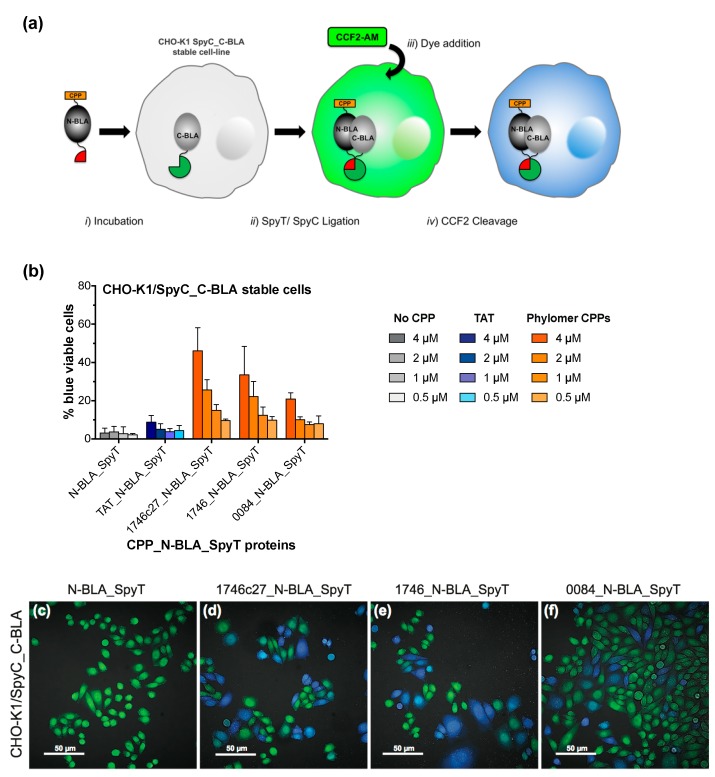
A CPP-mediated split β-lactamase cytosolic delivery assay. (**a**) Live-cell functional uptake can be measured when N-terminal β-lactamase (N-BLA), fused to a CPP sequence, penetrates the cell membrane to complement C-terminal β-lactamase protein (C-BLA) in the cytosol (step *i*). Expression of the split β-lactamase moieties as fusions with either SpyTag (CPP_N-BLA_SpyT) or SpyCatcher (SpyC_C-BLA) facilitates rapid formation of functional β-lactamase proteins through SpyT/SpyC ligation inside the cells (step *ii*). This internalization is detected after addition of dye substrate (step *iii*) through cleavage of cytosol-retained CCF2 (step *iv*), which is measured via a change in fluorescence signal from green (520 nm) to blue (450 nm). (**b**) Internalization of CPP_N-BLA_SpyT and β-lactamase complementation in CHO-K1/SpyC_C-BLA cells is dose-dependent, measured by CCF2 cleavage (increase in % blue viable cells as estimated by flow cytometry). N-BLA_SpyT protein (No CPP negative control) shows a negligible effect on the β-lactamase complementation signal over cell-line background at all concentrations tested. Error bars represent standard error of the mean of two independent experiments (duplicate samples). (**c**–**f**) Live cell confocal microscopy visually confirms the CPP-mediated cytosolic delivery of CPP_N-BLA_SpyT (4 µM) in CHO-K1/SpyC_C-BLA cells and subsequent β-lactamase complementation signal. N-BLA_SpyT protein (**c**, negative control) shows minimal evidence of internalization. β-Lactamase activity is dependent on cytosolic delivery of N-BLA_SpyT with phylomer CPPs 1746c27_N-BLA_SpyT (**d**)**,** 1746_N-BLA_SpyT (**e**), or 0084_N-BLA_SpyT (**f**). Bar scale is 50 µm.

In brief, CPP sequences are made as fusions to N-BLA_SpyT (CPP_N-BLA_SpyT, Figure 3a, step *i*). These proteins are incubated with mammalian cells stably expressing SpyC_C-BLA. β-Lactamase complementation, facilitated by SpyT/SpyC ligation, can occur in the cytosol (Figure 3a, step *ii*). Cells are washed to remove excess protein and then loaded with CCF2-AM cell-permeable dye for 1 h (Figure 3a, step *iii*). Once inside the cell, CCF2-AM is converted by endogenous cytoplasmic esterases to cytosol-retained CCF2. Cell penetrating peptide-dependent β-lactamase complementation and activity is detected by cleavage of the CCF2 substrate (Figure 3a, step *iv*). As in the full-length β-lactamase internalization assay, this reaction is measured as a shift in fluorescent signal from green to blue. Signal conversion continues as long as there is CCF2 substrate remaining inside the cell. The assay background was minimalized by ablating the *Amp*^R^ selection marker of the plasmid used to create the stable cell line and replacing it with *Kan*^R^.

To validate the split β-lactamase cytosolic delivery assay, we incubated TAT or phylomer CPP_N-BLA_SpyT proteins (sequences in Table A2) with CHO-K1/SpyC_C-BLA stable cells, measuring the fluorescence shift as percentage of blue fluorescent cells after 1 h (Figure 3b). Successful complementation of β-lactamase was dose- and CPP-dependent, with a limit of detection of ≤1 µM of CPP_N-BLA_SpyT. β-Lactamase complementation and N-BLA fragment internalization was visually confirmed using live cell confocal microscopy in CHO-K1/SpyC_C-BLA cells treated with Phylomer CPP_N-BLA_SpyT proteins (Figure 3c–f). The fluorescence signal is detectable as early as 30 min (Figure S2a). Signal conversion over time was confirmed by imaging cells treated with 1746c27_N-BLA_SpyT at sequential time points (Figure S2b,c), thus completing validation of the split assay system.

## 3. Discussion

Identifying and characterizing peptide sequences that efficiently internalize into cells is an ongoing focus of the CPP research field. Here, we present two complementary cell-based assays based on the uptake of a bacterial β-lactamase enzyme that can facilitate and streamline such efforts. Typically, β-lactamases cleave β-lactam antibiotics and thus confer antibiotic resistance to the bacteria [23,24]. However, in combination with fluorescent substrates such as CCF2-AM, β-lactamase becomes a versatile and robust reporter in mammalian cells.

The β-lactamase internalization and cytoplasmic delivery assays detailed in this report are robust, reproducible and rapid. β-lactamase uptake is detectable within 30 min, and the signal continues to convert as long as substrate is available. This improves assay sensitivity, which can be limited by photobleaching if fluorescence excitation is prolonged. This improved sensitivity is reflected in the concentration ranges used in assay validation (0.5–4 µM) and the sub-micromolar limit of detection for both β-lactamase CPP assays. Such concentrations are well below the high protein concentrations (>20 µM) where cationic peptides may non-specifically internalize or “flood” into cells [25].

The β-lactamase assays are highly versatile. First, they can be used to assess any synthesized or expressed CPP and should be compatible with cell targeting motifs such as tripeptide Arg-Gly-Asp (RGD, integrin-binding) and CPPs using different internalization mechanisms. Second, β-lactamase internalization is compatible with any cell type that can take up the CCF2-AM substrate. Moreover, CPP-delivery of the same cargo can be assessed across multiple cell lines in parallel. The split β-lactamase assay is also compatible with any cell line amenable to transfection of SpyC_C-BLA. However, using a stable cell line instead of a transient line improves the assay throughput and sensitivity. Third, the scalability of these assays makes them amenable to high-throughput formats, robotics, and imaging techniques. Fourth, these assays could also be used as reporter assays in assessing different uptake mechanisms, for example with inclusion of various endocytic inhibitors or measurements at earlier time points. Fifth, using SpyT/SpyC ligation also allows the β-lactamase assays to access synthetic chemistry for biological validation of CPPs. For example, synthetic CPPs made from L- and/or D-amino acids may be conjugated to β-lactamase to explore the effect of improved CPP protease resistance [26,27,28].

We suggest these two assays have complementary strengths. The full-length β-lactamase assay assesses uptake and delivery via CPP in the context of a biologically functional cargo. It has particular utility for high-throughput assessment of CPP activity by conjugating different CPPs to the same SpyC_BLA cargo. Identified, potent sequences can then be assessed in more detail, examining uptake at low concentrations using recombinant CPP_BLA proteins and proof of cytosolic delivery using β-lactamase complementation. Using a protein cargo such as β-lactamase is a better approximation than a small molecule fluorophore for the delivery of a therapeutic biologic. Together, the complementary assays can be used as a platform for versatile, rapid evaluation of CPP activity and proof of cytoplasmic delivery, which should prove valuable in the exploration of what makes a good CPP.

## 4. Materials and Methods

### 4.1. Mammalian Cell Culture

The CHO-K1 cell line (Chinese hamster ovary) was obtained from American Type Culture Collection (ATCC, Manassas, VA, USA); the T47D cell line (human breast cancer) was provided by Dr. P. Dallas, Telethon Kids Institute (Subiaco, WA, Australia). Cell lines were maintained in a humidified incubator at 37 °C with 5% CO_2_. CHO-K1 and T47D cells were cultured in RPMI 1640 (Gibco, Thermo Fisher Scientific, Waltham, MA, USA) supplemented with 10% heat-inactivated fetal calf serum (FCS), 2 mM Glutamax, 100 U/mL penicillin-streptomycin.

The CHO-K1/SpyC_C-BLA monoclonal stable cell line was made using FlpIn™ technology (Flp-In™-CHO Cell Line, Invitrogen, Carlsbad, CA, USA). To make the FlpIn plasmid for stable transformation, a coding sequence for SpyC_C-BLA fusion protein (Table A2) was synthesized (ATUM) and cloned into the *BamH*I and *Xho*I sites of pcDNA5/FRT (Invitrogen). Then the *Amp^R^* gene was excised from the plasmid backbone, using *Bgl*II and *BspH*I restriction enzymes (New England Biolabs (NEB), Ipswich, MA, USA), and replaced with a *Kan^R^* cassette cloned via *Nco*I and *Bgl*II (NEB). Stable cell line CHO-K1/SpyC_C-BLA was cultured in HAM’s F12-K (Kaighn) medium (Gibco), supplemented with 10% heat-inactivated FCS (Rowe Scientific, Minto, NSW, Australia), 2 mM Glutamax, 100 U/mL penicillin-streptomycin, and 800 µg/mL Hygromycin B (Gibco).

### 4.2. β-Lactamase Fluorogenic Assay

Cells were seeded in 24-well plates (CHO-K1, 100,000 cells/well; T47D 100,000 cells/well) in complete medium. Cells were incubated overnight to allow for cell adherence. On the day of the assay, cells were incubated with SpyC_BLA or SpyC_BLA/CPP_SpyT conjugates at 37 °C, 5% CO_2_ for 1 h. After incubation, the conjugate/protein-containing media was removed by aspiration and the cells were washed once with phosphate buffered saline (PBS) then detached from the plates by incubation with 0.25% Trypsin-EDTA (Gibco) solution for 5 min at 37 °C. Trypsination was stopped by the addition of complete media and cells dislodged by pipetting; collected cells were transferred to 96-well plates and washed once with Hank’s balanced salt solution (HBSS, Gibco). CCF2-AM solution was prepared according to the enhanced loading protocol as outlined in the manufacturers specifications (GeneBLAzer™ In Vivo Detection Kit, Thermo Fisher Scientific, Waltham, MA, USA); Hank’s balanced salt solution was used instead of GeneBLAzer™ In Vivo Detection Kit Solution C. CCF2-AM was applied to CHO-K1 or T47D cells at 2 µM or 1 µM final concentration, respectively. CCF2-AM solution was then removed, the cells were resuspended in FACS buffer (HBSS + 1% bovine serum albumin, BSA), and then measured by flow cytometry (BD Biosciences Fortessa with FACSDiva software (FACSDiva v8, BD Biosciences Australia, North Ryde, NSW, Australia), or Attune NxT flow cytometers (Thermo Fisher Scientific). Fortessa flow cytometry used excitation with a 405 nm violet laser, and detection with BV510 (525/50) and V450 (450/50) filters; Attune flow cytometry used excitation with a 405 nm violet laser, and detection with 512/25 and 440/50 filters. Data were analyzed with FlowJo X 10.4.1 software (Tree Star Inc., v10.4.1, Ashland, OR, USA); dot plots for gating strategies are shown (Figure S3). The percentages of β-lactamase positive cells for each sample were graphed against the concentration of protein added to the cells, using Prism (version 7.0a, GraphPad, La Jolla, CA, USA).

### 4.3. Confocal Live-Cell Microscopy

Cells were seeded (CHO-K1, 37,500 cells/mL; T47D 37,500 cells/mL) onto 4-well ibiTreat chamber slides (Thermo Fisher Scientific) and incubated for 48 h at 37 °C, 5% CO_2_. The medium was removed and replaced with medium containing SpyC_BLA/CPP_SpyT conjugated or unconjugated SpyC_BLA proteins and cells were incubated for 2 h at 37 °C, 5% CO_2_. After incubation, the cells were washed to remove any SpyC_BLA protein that had not internalized, loaded with substrate CCF2-AM (2 µM final concentration in media containing 1% FCS), and incubated for 1 h at room temperature. Live cells were visualized through confocal microscopy. Images were collected using a Nikon C2 Confocal Microscope through a 40× differential interference contrast (DIC) objective after excitation by a 408 nm laser. Bar scales in images represent 50 µm or 14.6 µm (zoom magnification 3.4×).

### 4.4. Peptides

CPP_SpyTag synthetic peptides (Pepscan GmbH, Lelystad, The Netherlands, and Mimotopes, Mulgrave, VIC, Australia; sequences in Table A1) were conjugated to SpyC_BLA proteins at a peptide:protein ratio of 1.125:1 or 1.25:1, with a 50 µM final concentration for the SpyC_BLA protein. Reactions were performed in 50 mM HEPES (4-(2-hydroxyethyl)-1-piperazineethanesulfonic acid), 200 mM NaCl (pH 7.0) and incubated for 2 h at room temperature with gentle mixing and/or or left overnight at 4 °C. Conjugation efficiencies were analyzed on 4–12% NuPAGE Bis-Tris protein gels stained with SimplyBlue^TM^ Safe Stain (Invitrogen).

### 4.5. Recombinant Protein Expression and Purifcation

DNA sequences of BLA fusion proteins (Table A2) were synthesized and cloned (ATUM, Newark, CA, USA) into the *Nco*I and *Xho*I sites of the pET28a+ protein expression vector (Merck Millipore, Burlington, MA, USA).

Recombinant SpyC_BLA protein was expressed as His6-N-terminally tagged fusion in *E. coli* strain BL21 (DE3) Gold (Agilent Technologies, Santa Clara, CA, USA), purified using IMAC and filter-sterilized as previously described [14], with an additional size exclusion chromatography (SEC) purification step.

In brief, the cell pellet was lysed by sonication in 50 mM HEPES, 200 mM NaCl, pH 7.0, purified by IMAC (HisTrapHP™ GE Healthcare, Chicago, IL, USA) with gradient elution (AKTAxpress, GE Healthcare) with 20–500 mM imidazole. Proteins were then subject to an additional size exclusion chromatography (Sephacryl HiPrep™ 26/60 S100 and Sephacryl HiPrep™ 26/60 S200, GE Healthcare) purification in 50 mM HEPES, 200 mM NaCl, pH 7.0 (AKTAxpress), concentrated (Jumbosep-10K spin columns, PALL Life Sciences, Port Washington, NY, USA) and filter sterilized. Protein purity was confirmed by analysis on 4–20% Mini-PROTEAN TGX stain-free™ gel (BioRad Australia, Gladesville, NSW, Australia) stained with gel code blue stain (Thermo Fisher Scientific). Protein concentration was determined by bicinchoninic acid (BCA) assay (Pierce™ BCA Protein Assay Kit, Thermo Fisher Scientific).

Recombinant CPP_BLA proteins were expressed as His6-tagged fusions in *E. coli* strain BL21(DE3) Gold (Agilent Technologies), lysed by sonication in 20 mM sodium phosphate, 250 mM NaCl, pH 7.0, and purified by IMAC (Phytip IMAC columns, Phynexus, San Jose, CA, USA). Eluted proteins were desalted (PD10 columns, Merck Millipore), concentrated (Amicon-10K spin columns, Merck Millipore) and filter sterilized. Protein purity was confirmed by analysis on 4–12% SDS-PAGE (sodium dodecyl sulfate–polyacrylamide gel electrophoresis) stained with SimplyBlue^TM^ SafeStain (Thermo Fisher Scientifc). Protein concentration was determined by BCA assay (Pierce™ BCA Protein Assay Kit, Thermo Fisher Scientific).

Recombinant CPP_N-BLA_SpyT proteins were expressed as His6-tagged fusions in *E. coli* strain BL21(DE3) Gold (Agilent Technologies). Proteins were extracted from inclusion bodies by dissolution in 8 M Urea, 20 mM sodium phosphate, 500 mM NaCl, pH 7.0, and purified by IMAC (Phytip IMAC columns, Phynexus). Eluted protein was step-wise dialyzed into 20 mM sodium phosphate, 250 mM NaCl, pH 7.0, concentrated (Amicon-10K spin columns, Merk Millipore), and filter sterilized. Protein purity was confirmed by analysis on 4–12% SDS-PAGE stained with SimplyBlue^TM^ SafeStain. Protein concentration was determined by BCA assay (Pierce™ BCA Protein Assay Kit, Thermo Fisher Scientific).

## Supplementary Materials

The following are available online at http://www.mdpi.com/2218-273X/8/3/51/s1, Figure S1: Verification of CPP-mediated β-lactamase internalization by immunoblotting, Figure S2: Split β-lactamase assay time course, Figure S3: Flow cytometry gating strategies for CHO-K1 and T47D cells in the β-lactamase assays, Supplementary Methods, Supplementary References ([29,30]).

## Appendix A

**Table A1:** CPP_SpyT peptide sequences.

Name	Sequence ^1^	Length	Net Charge (pH 7)
TAT_SpyT	GRKKRRQRRRGASAHIVMVDAYKPTKG	27	9+
1746c27_SpyT	KKKKQPPKPKKPKTQEKKKKQPPKPKRGASAHIVMVDAYKPTKG	44	15+
1746_SpyT	PLKPKKPKTQEKKKKQPPKPKKPKTQEKKKKQPPKPKRGASAHIVMVDAYKPTKG	55	18+
0031_SpyT	RASPSEQRRKRRRCHRGETQRPDFEAEIEKQQRGASAHIVMVDAYKPTKG	51	6+
0084_SpyT	RKQKSLQTKLAENPPVPRKKRQSRPRWKQWLQKGASAHIVMVDAYKPTKG	50	12+
0167_SpyT	PSSTPATNVARPRLNPIRGHKFALAVPNSRTRGASAHIVMVDAYKPTKG	49	7+
0169_SpyT	PLTQRTLQRGKKPKQRQNWKKARTSSAKTAPKTVVSRTTSQRKGASAHIVMVDAYKPTKG	60	15+
1788_SpyT	LFVDKATPQIYYTPSESVTVKSKGKNRRKKSGASAHIVMVDAYKPTKG	48	7+
1869_SpyT	PPHPRPLPAPAQSRKKQKGRAGRGHEKTGASVLRGPQKPHPLPAQLRGASAHIVMVDAYKPTKG	64	11+

^1^ CPP Sequence in orange; SpyT sequence in green.

**Table A2:** SpyCatcher β-lactamase fusion proteins.

Name	Sequence ^1^	kDa	pI ^2^
SpyC_BLA	MGHHHHHHGATLEVLFQGPGGSGSDSATHIKFSKRDEDGKELAGATMELRDSSGKTISTWISDGQVKDFYLYPGKYTFVETAAPDGYEVATAITFTVNEQGQVTVNGKATKGSHPETLVKVKDAEDQLGARVGYIELDLNSGKILESFRPEERFPMMSTFKVLLCGAVLSRIDAGQEQLGRRIHYSQNDLVEYSPVTEKHLTDGMTVRELCSAAITMSDNTAANLLLTTIGGPKELTAFLHNMGDHVTRLDRWEPELNEAIPNDERDTTMPAAMATTLRKLLTGELLTLASRQQLIDWMEADKVAGPLLRSALPAGWFIADKSGAGERGSRGIIAALGPDGKPSRIVVIYTTGSQATMDERNRQIAEIGASLIKHWQLGSASGTTGATSGEF	42.4 kDa	5.6
TAT_BLA	MASGRKKRRQRRRASSGSHHHHHHGATLEVLFQGPGGSHPETLVKVKDAEDQLGARVGYIELDLNSGKILESFRPEERFPMMSTFKVLLCGAVLSRIDAGQEQLGRRIHYSQNDLVEYSPVTEKHLTDGMTVRELCSAAITMSDNTAANLLLTTIGGPKELTAFLHNMGDHVTRLDRWEPELNEAIPNDERDTTTPAAMATTLRKLLTGELLTLASRQQLIDWMEADKVAGPLLRSALPAGWFIADKSGAGERGSRGIIAALGPDGKPSRIVVIYTTGSQATMDERNRQIAEIGASLIKHWQLGSASGTTGATSG	34.3 kDa	7.2
1746c27_BLA	MASKKKKQPPKPKKPKTQEKKKKQPPKPKRASSGSHHHHHHGATLEVLFQGPGGSHPETLVKVKDAEDQLGARVGYIELDLNSGKILESFRPEERFPMMSTFKVLLCGAVLSRIDAGQEQLGRRIHYSQNDLVEYSPVTEKHLTDGMTVRELCSAAITMSDNTAANLLLTTIGGPKELTAFLHNMGDHVTRLDRWEPELNEAIPNDERDTTTPAAMATTLRKLLTGELLTLASRQQLIDWMEADKVAGPLLRSALPAGWFIADKSGAGERGSRGIIAALGPDGKPSRIVVIYTTGSQATMDERNRQIAEIGASLIKHWQLGSASGTTGATSG	36.1 kDa	9.2
1746_BLA	MASPLKPKKPKTQEKKKKQPPKPKKPKTQEKKKKQPPKPKRASSGSHHHHHHGATLEVLFQGPGGSHPETLVKVKDAEDQLGARVGYIELDLNSGKILESFRPEERFPMMSTFKVLLCGAVLSRIDAGQEQLGRRIHYSQNDLVEYSPVTEKHLTDGMTVRELCSAAITMSDNTAANLLLTTIGGPKELTAFLHNMGDHVTRLDRWEPELNEAIPNDERDTTTPAAMATTLRKLLTGELLTLASRQQLIDWMEADKVAGPLLRSALPAGWFIADKSGAGERGSRGIIAALGPDGKPSRIVVIYTTGSQATMDERNRQIAEIGASLIKHWQLGSASGTTGATSG	37.4 kDa	9.4
N-BLA_SpyT	MGHHHHHHGATLEVLFQGPGGSQLHPETLVKVKDAEDQLGARVGYIELDLNSGKILESFRPEERFPMMSTFKVLLCGAVLSRIDAGQEQLGRRIHYSQNDLVEYSPVTEKHLTDGMTVRELCSAAITMSDNTAANLLLTTIGGPKELTAFLHNMGDHVTRLDRWEPELNEAIPNDERDTTMPAAMATTLRKLLTGGGSASGGSGTGATSGAHIVMVDAYKPTKGVDG	24.5 kDa	5.7
TAT_N-BLA_SpyT	MASGRKKRRQRRRASSGSHHHHHHGTGAGSGHPETLVKVKDAEDQLGARVGYIELDLNSGKILESFRPEERFPMMSTFKVLLCGAVLSRIDAGQEQLGRRIHYSQNDLVEYSPVTEKHLTDGMTVRELCSAAITMSDNTAANLLLTTIGGPKELTAFLHNMGDHVTRLDRWEPELNEAIPNDERDTTTPAAMATTLRKLLTGGGSASGGSGTGATSGAHIVMVDAYKPTKGGTGGS	25.3 kDa	7.9
1746c27_N-BLA_SpyT	MASKKKKQPPKPKKPKTQEKKKKQPPKPKRASSGSHHHHHHGTGAGSGHPETLVKVKDAEDQLGARVGYIELDLNSGKILESFRPEERFPMMSTFKVLLCGAVLSRIDAGQEQLGRRIHYSQNDLVEYSPVTEKHLTDGMTVRELCSAAITMSDNTAANLLLTTIGGPKELTAFLHNMGDHVTRLDRWEPELNEAIPNDERDTTTPAAMATTLRKLLTGGGSASGGSGTGATSGAHIVMVDAYKPTKGGTGGS	27.2 kDa	9.3
1746_N-BLA_SpyT	MASPLKPKKPKTQEKKKKQPPKPKKPKTQEKKKKQPPKPKRASSGSHHHHHHGTGAGSGHPETLVKVKDAEDQLGARVGYIELDLNSGKILESFRPEERFPMMSTFKVLLCGAVLSRIDAGQEQLGRRIHYSQNDLVEYSPVTEKHLTDGMTVRELCSAAITMSDNTAANLLLTTIGGPKELTAFLHNMGDHVTRLDRWEPELNEAIPNDERDTTTPAAMATTLRKLLTGGGSASGGSGTGATSGAHIVMVDAYKPTKGGTGGS	28.5 kDa	9.5
0084_N-BLA_SpyT	MASRKQKSLQTKLAENPPVPRKKRQSRPRWKQWLQKASSGSHHHHHHGTGAGSGHPETLVKVKDAEDQLGARVGYIELDLNSGKILESFRPEERFPMMSTFKVLLCGAVLSRIDAGQEQLGRRIHYSQNDLVEYSPVTEKHLTDGMTVRELCSAAITMSDNTAANLLLTTIGGPKELTAFLHNMGDHVTRLDRWEPELNEAIPNDERDTTTPAAMATTLRKLLTGGGSASGGSGTGATSGAHIVMVDAYKPTKGGTGGS	28.1 kDa	9.0
SpyC_C-BLA	MGGSQLDSATHIKFSKRDEDGKELAGATMELRDSSGKTISTWISDGQVKDFYLYPGKYTFVETAAPDGYEVATAITFTVNEQGQVTVNGKATKGGGSASGTGGSGTGATSGLLTLASRQQLIDWMEADKVAGPLLRSALPAGWFIADKSGAGERGSRGIIAALGPDGKPSRIVVIYTTGSQATMDERNRQIAEIGASLIKHWVDG	21.4 kDa	5.7

^1^ SpyCatcher sequence in light blue; BLA sequences in red; His tag used in purification in blue; cell penetrating peptide (CPP) sequence in orange; SpyT sequence in green. ^2^ Isoelectric point (pI).
