# Supplementary material for: β-Lactamase Tools for Establishing Cell Internalization and Cytosolic Delivery of Cell Penetrating Peptides
Source: Biomolecules. 2018 Jul 11;8(3):51. doi: 10.3390/biom8030051 (PMC6163455; doi:10.3390/biom8030051)
Supplement: Supplementary file 1 [file biomolecules-08-00051-s001.pdf]

biomolecules

MDPI

Supplementary Information

Supplementary Figures: β-Lactamase Tools for Establishing Cell Internalization and Cytosolic Delivery of Cell Penetrating Peptides

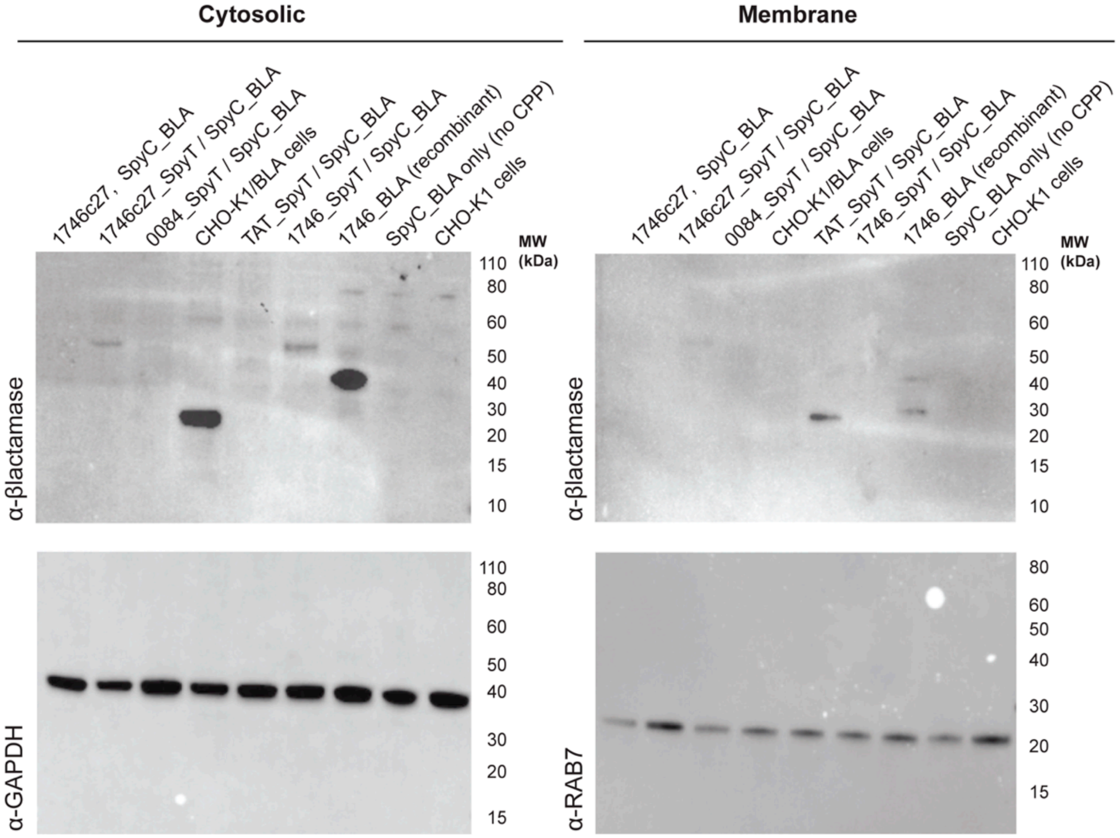

Figure S1. Verification of cell penetrating peptides (CPP)-mediated β-lactamase internalization by immunoblotting. MW: molecular weight.

Aliquots (4 μg) of cytosolic and membrane soluble CHO-K1 cell fractions from β-lactamase assays were separated by sodium dodecyl sulfate–polyacrylamide gel electrophoresis (SDS-PAGE) through a 4-12% (*w*/*v*) polyacrylamide Bis-Tris gel, transferred to polyvinylidene difluoride (PVDF) membrane, and immunoblotted. β-lactamase conjugated (e.g., 1746_SpyT/SpyC_BLA) or fused (e.g., 1746_BLA recombinant) to 1746c27 and 1746 Phylomer cell penetrating peptides (CPPs) was detected in the cytosolic fraction to varying extents, while TAT-conjugated β-lactamase was predominantly detected in the membrane fraction of cell lysates. GAPDH (37 kDa) expression was used to show comparable loading for cytosolic fractions while endosomal marker Rab7 (23 kDa) protein expression was used to show comparable loading for membrane fractions. In agreement with the uptake assays, recombinant 1746-BLA showed a very strong signal in the cytosolic fractions, with a relatively small amount retained in membrane fractions. This indicates that the majority of internalized 1746_BLA is delivered into the cytosol by the 1746 CPP. Internalization of unconjugated SpyC_BLA protein was not detected, either on its own (SpyC_BLA (no CPP)) or in the presence of 1746c27 peptide (1746c27, SpyC_BLA), illustrating there is no bystander uptake effect.

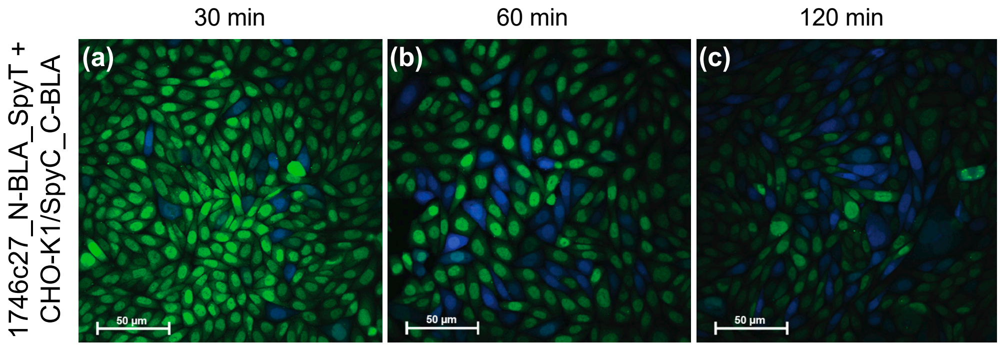

Figure S2. Split β-lactamase assay time course.

Live cell confocal microscopy imaging the CPP-mediated cytosolic internalization of 1746c27_N-BLA_SpyT (8 µM) in CHO-K1/C-BLA cells at **(a)** 30 min, **(b)** 60 min, and **(c)** 120 min after addition of CCF2-AM dye. The β-lactamase complementation signal is detectable as early as 30 min and continues to convert over time. Repeated excitation over time results in photobleaching of the converted fluorescence resonance energy transfer (FRET) substrate. Bar scale is 50 µm.

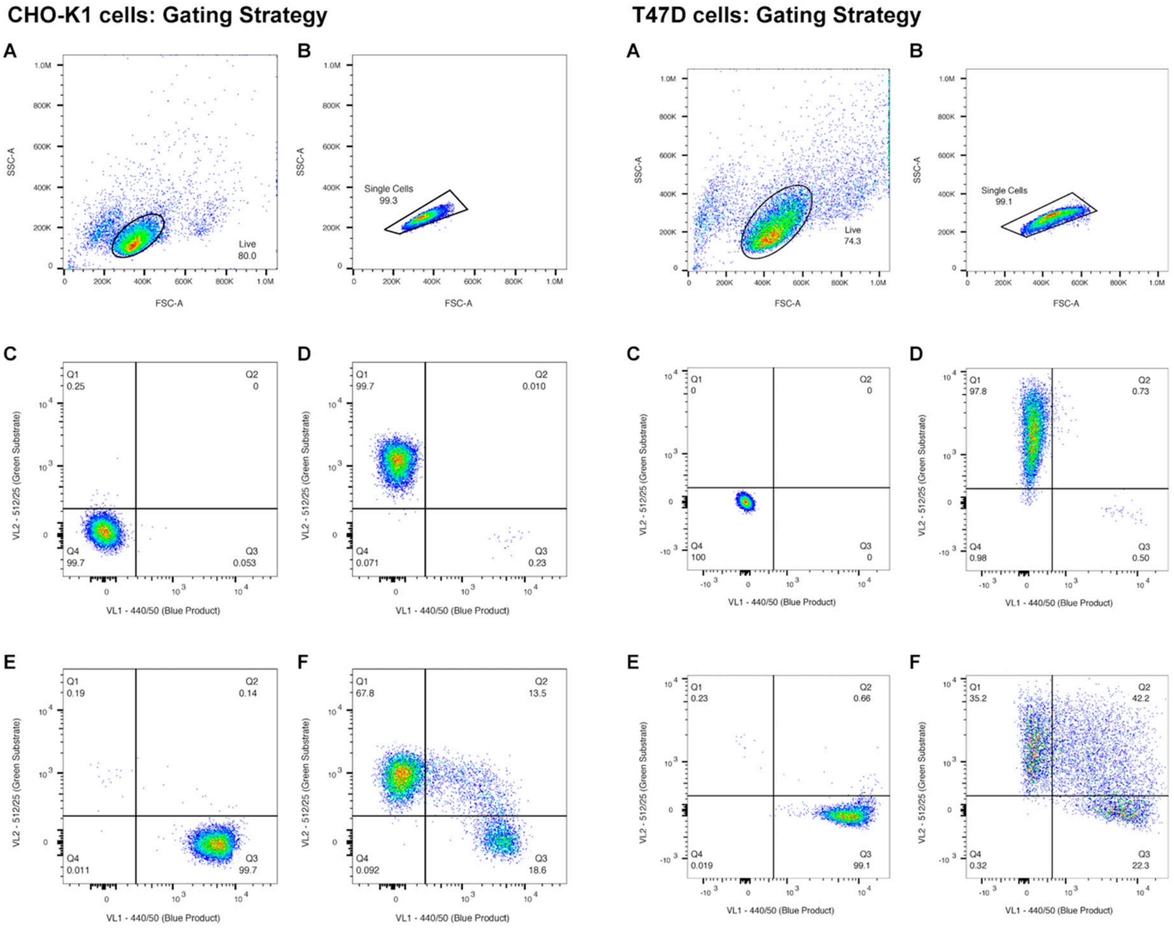

Figure S3. Flow cytometry gating strategies for CHO-K1 and T47D cells in the β-lactamase assays.

Flow cytometry gating was performed in the following order (**A-F**) for both CHO-K1 and T47D cells: (**A**) FSC-A/SSC-A–Gating for whole cells. Dead cells and other debris are excluded; (**B**) FSC-A/FSC-H–Gating for single cells; (**C**) Double negative population–CHO-K1 cells with no CCF2-AM addition; (**D**) Substrate positive population–CCF2-AM positive cells; (**E**) Product positive

population– CHOFlpIn TEM-1 cells expressing beta-lactamase TEM-1, which convert all available CCF2-AM into blue product; (**F**) 1746c27-TEM-1 peptide conjugate (4 μM) for 1 h prior to CCF2-AM loading.

Supplementary Methods

Cell fractionation

CHO-K1 cells were seeded in 6-well plates (1×10^6 cells/well). The following day, cells were incubated with SpyC_BLA or SpyC_BLA/CPP_SpyT conjugates (8 μM) at 37 °C/ 5% CO_2 for 1.5 h, then washed and detached from the plates by incubation with trypsin for 5 min. Cells were washed again and then sequentially lysed; cytosolic and membrane soluble cell fractions were prepared using digitonin (100 μg/mL) in Lysis buffer A for complete cytosol extraction, and Lysis buffer B for membrane extraction, respectively [29,30]. Protein concentrations were quantitated (Pierce™ BCA protein assay kit and Micro BCA™ protein assay kit, Thermo Fisher Scientific, Watham, MA, USA) for cytosolic and membrane fractions, respectively.

Immunoblotting

Aliquots of extracted fractions (4 μg) were separated through a 4–12% Bis-Tris gel (Invitrogen, Carlsbad, CA, USA). Protein expression in the fractions was detected using monoclonal anti-β-lactamase antibody [8A5.A10] (Abcam; cat #12251, Lot#: GR232253-13; diluted 1:500), polyclonal anti-Rab7 antibody (Sigma Aldrich Australia, Castle Hill, NSW, Australia; cat #R4779-200 uL, lot #016M4839V; diluted 1:1000), or polyclonal anti-GAPDH antibody (Thermo Fisher Scientific; cat #PA1-987, lot #SC247984; diluted 1:5000), followed by anti-mouse (Thermo Fisher Scientific; cat #31430, lot #SF252846; diluted 1:50000) or anti-rabbit (Thermo Fisher Scientific; cat #A27036, lot #SE247011A; diluted 1:20000) secondary antibodies. After detection using SuperSignal West maximum sensitivity substrate (Thermo Scientific), images were captured on a ChemiDoc Gel Imaging System (BioRad Australia, Gladesville, NSW, Australia).

References

29. Baghirova, S.; Hughes, B. G.; Hendzel, M. J.; Schulz, R. Sequential fractionation and isolation of subcellular proteins from tissue or cultured cells. *MethodsX* **2015**, *2*, 440–445.
30. Holden, P.; Horton, W. A. Crude subcellular fractionation of cultured mammalian cell lines. *BMC Research Notes* **2009**, *2*, 243.

© 2018 by the authors. Submitted for possible open access publication under the terms and conditions of the Creative Commons Attribution (CC BY) license (http://creativecommons.org/licenses/by/4.0/).
